# In vitro study on human cytomegalovirus affecting early pregnancy villous EVT's invasion function

**DOI:** 10.1186/1743-422X-8-114

**Published:** 2011-03-11

**Authors:** Liu Tao, Chen Suhua, Chen Juanjuan, Yin Zongzhi, Xiao Juan, Zhang Dandan

**Affiliations:** 1 Department of Obsterics and Gynecology, Tongji Hospital, Tongji Medical College, Huazhong University of Science and Technology, Wuhan, China; 2 Department of Obsterics and Gynecology, Tai'an City Central Hospital, Tai'an, China

## Abstract

**Background:**

Human cytomegalovirus (HCMV) is the most common pathogen in uterus during pregnancy, which may lead to some serious results such as miscarriage, stillbirth, cerebellar malformation, fetus developmental retardation, but its pathogenesis has not been fully explained. The hypofunction of extravillous cytotrophoblast (EVT) invasion is the essential pathologic base of some complications of pregnancy. c-erbB-2 is a kind of oncogene protein and closely linked with embryogenesis, tissue repair and regeneration. Matrix metalloproteinase (MMP) is one of the key enzymes which affect EVT migration and invasion function. The expression level changes of c-erbB-2, MMP-2 and MMP-9 can reflect the changes of EVT invasion function.

**Results:**

To explore the influence of HCMV on the invasion function of EVT, we tested the protein expression level changes of c-erbB-2, MMP-2 and MMP-9 in villous explant cultured in vitro infected by HCMV, with the use of immunohistochemistry SP method and western blot. We confirmed that HCMV can reproduce and spread in early pregnancy villus; c-erbB-2 protein mainly expressed in normal early pregnancy villous syncytiotrophoblast (ST) remote plasma membrane and EVT, especially remote EVT cell membrane in villous stem cell column, little expressed in ST proximal end cell membrane and interstitial cells; MMP-2 protein primarily expressed in early pregnancy villous EVT endochylema and rarely in villous trophoblast (VT), ST and interstitial cells; MMP-9 protein largely expressed in early pregnancy villous mesenchyme, EVT and VT endochylema. Compared with control group, the three kinds of protein expression level in early pregnancy villus of virus group significantly decreased (P < 0.05).

**Conclusion:**

HCMV can infect villus in vitro and cause the decrease of early pregnancy villous EVT's invasion function.

## Introduction

Human cytomegalovirus (HCMV) is the most common pathogen in uterus during pregnancy, which may lead to some serious results such as miscarriage, stillbirth, cerebellar malformation, fetus developmental retardation, but its pathogenesis has not been fully explained[1]. It is currently considered that in later stage of blastula's nidation, cytotrophoblast (CT) differentiates to villous trophoblast (VT) and extravillous cytotrophoblast (EVT). VT is active in karyokinesis, fusing to form syncytiotrophoblast (ST). ST performs the functions of internal secretion, immunity and material exchange and so on; EVT forms cell column after its proliferation and migration, invasively grow towards uterus mesenchyme and spiral artery lumen, rebuilding spiral artery[2]. The hypofunction of EVT invasion is the essential pathologic base of miscarriage, premature birth, stillbirth, fetus developmental retardation, gestational hypertension and other complications of pregnancy[3]. As being one of the symbolic proteins of EVT, c-erbB-2 protein participates in the process of EVT invasion[4]. In addition, Matrix metalloproteinase (MMP) is one of the key enzymes which affect EVT migration and invasion function, especially MMP-2 and MMP-9. At present, there are only a few studies on HCMV's influence on EVT invasion function. In order to explore the correlation of EVT invasion dysfunction and HCMV intrauterine infection which causes miscarriage, stillbirth and fetus developmental retardation, this paper adopts early pregnancy villous explant cultured in vitro, so as to observe HCMV's influence on EVT invasion function in villus. Here is the report:

## Materials and methods

### Materials

#### Specimen and sources of the virus

The tissue of villus is from healthy pregnant women whose peripheral blood is HCMV antibody negative and whose gestational age are between 5 to 10 weeks, who are voluntarily conducted abortion because of planned parenthood, from department of gynecology and obstetrics of Tongji Hospital of Tongji Medical College of Huazhong University of Science and Technology from March 2010 to July 2010. The research procedure complies with the ethnical standard drew by Ethics Committee of Tongji Hospital of Tongji Medical College. HCMV AD169 strain is provided by Hubei Province Institute of Viruses, while TCID_50 _is 10^-5^.

#### Sources of main reagents

DMEM/Ham's F 12 culture medium (Gibco), standard fetal bovine serum (Hyclone), rabbit anti HCMV pp65 polyclonal antibody (Santa), rabbit anti c-erbB-2 polyclonal antibody, rabbit anti MMP-2 and MMP-9 polyclonal antibodies (Wuhan Boster Bio-engineering Co., Ltd), SP immunohistochemistry kit (Zhongshan Goldenbridge Biotechnology Co., Ltd), total protein extraction kit (BestBio), β-actin loading control antibody (Beijing Biosynthesis Biotechnology Co., Ltd) and so on.

### Methods

#### Villous explant culture and experimental grouping

Improvements are made by referring to Dong cultural method[5]. The specific method is as follow: villous tissue was rapidly taken to laboratory by being placed in sterile pre-cooling D-hank's solution (containing 100 UI/ml penicillin and 100 μg/ml streptomycin), which were washed for three times for removing gore and non-cruor; it was put into serum-free DMEM/Ham's F 12 culture medium, and the apical tissue of villus (wet weight 40~50 mg) was carefully cut and inoculated in 24-hole cell culture plate, being cultured overnight in DMEM/Ham's F 12 culture medium containing 10% fetal bovine serum with 5% CO_2 _at 37°C; after throwing away the supernatant, it was changed to be DMEM/Ham's F 12 culture medium containing 3% fetal bovine serum. In infection group, 1 ul HCMV virus solution was added in every hole, while equal amount of PBS was added into every hole in control group; culture medium was absorbed, threw away in the two groups above after 2 h culture and washed by PBS for twice and added into complete medium; after 48 h, villous explants were taken out. Total protein was extracted from part of villous tissue for Western blot detection. The rest of villous tissue was fixed for 24 h in formalin buffer solution and paraffin wax embedding sliced (thickness of slice was 3 um), for conducting pathological and immunohistochemical detection.

#### Hematoxylin eosin(HE) staining

Two groups of villous slice are being HE stained. Optical microscope is utilized to observe villus pathological changes.

#### Immunohistochemistry staining

Two groups of slices are taken to conduct conventional treatment. Immunohistochemistry streptavidin-perosidase(SP) method is used to detect HCMV pp65, c-erbB-2, MMP-2 and MMP-9, and the method please refers to reference[6]. Paraffin embedded sections were prepared in a microtome and de-paraffinized in a series of ethanols and xylenes. Sections were blocked with methanol containing 3% H_2_O_2_, sequentially 10% goat normal serum, and incubated with rabbit anti HCMV pp65 polyclonal antibody, rabbit anti c-erbB-2 polyclonal antibody, rabbit anti MMP-2 and MMP-9 polyclonal antibodies (1:100 dilution) overnight at 4°C. They were then sequentially treated with a biotinylated goat anti-rabbit antibody and a horseradish peroxidase(HRP) labelled streptoavidin for 1 h at 37°C respectively. Development was performed by treating the sections with a Liquid DAB-Plus Substrate kit. After counter-staining with haematoxylin, immunostaining of these proteins on the tissue sections was detected by light microscope. Under optical microscope, positive signals were nankeen or brown grains. HMIAS-2000 high definition color medical image analysis system was adopted to process images. Under high magnification, 10 visual fields were randomly observed, testing positive expression cells' average optical density for semi-quantitative statistical analysis.

Controls The above immunohistochemical procedures controls were performed replacing the primary antibody by PBS. Further controls were performed omitting the secondary antibody. The controls were always negative.

#### Western Blot detection

It is to detect c-erbB-2, MMP-2 and MMP-9 expression levels. Referring to the instruction of kits, two groups of villous total protein were extracted and stored in fridge at -70°C. Bradford method was used to measure protein concentration. In every sample, 50 μg 10% SDS-PAGE was taken and wet transferred to PVDF membrane. 5% skimmed milk powder was sealed for 2 h at room temperature. Rabbit anti c-erbB-2, MMP-2 and MMP-9 polyclonal antibodies were respectively added, cultured overnight at 4°C. After TBST washing membrane, it was added into second antibody and cultured for 2 h at room temperature. TBST washes membrane and ECL reagent developed color. Exposure imaging was done under gel imager. Quantityone gel image analysis software detected different groups' protein and their loading control β-actin protein's absorbance value, showing target protein level by the ratio of target strip and average absorbance of loading control protein.

### Statistical analysis

These experiments above were repeated 3 times. SPSS 13.0 statistical software is adopted. Value of number is expressed by the mean ± standard deviation. Differences among groups are conducted independent sample t testing, while P < 0.05 means the difference is of statistical significance.

## Results

### Villus HE staining

**Figure 1:**
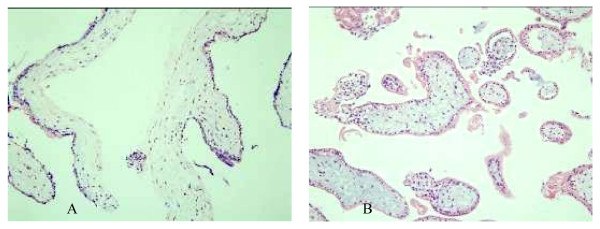
**Histology of two groups'villous explants under HE staining**. A. Human villous explants in vitro HE ×200; B. Human villous explants infected by HCMV in vitro HE ×200.

In control group, ST and CT were visible on villous surface, and cell boundary was clear. Villous interstitial substance was loose primitive interstitial substance. In interstitial substance, small blood vessels can be seen(Figure 1A). There was no obvious abnormality in villus of infected group(Figure 1B).

### Villus HCMV detection results

**Figure 2:**
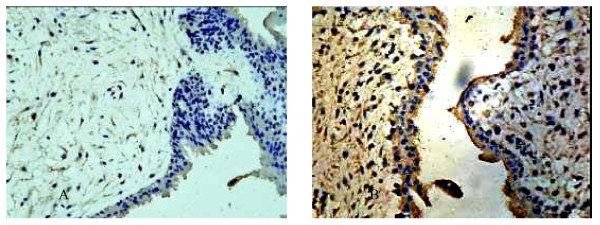
**The expression of HCMVpp65 antigen in two groups'villous explants detected by immunohistostaining**. A. The expression of HCMVpp65 antigen in normal human villous explants detected by immunohistostaining ×400; B. The expression of HCMVpp65 antigen in human villous explants infected by HCMV detected by immunohistostaining ×400.

Immunohistochemistry staining results were shown in Figure 2. When infected group was added into HCMV and cultured for 48 h, HCMVpp65 signals showed almost all CT, EVT, ST, interstitial cells and around villous interstitial substance small blood vessels in villus(Figure 2B), while there was no HCMVpp65 antigen signal in control group(Figure 2A). HCMV was added into early pregnancy villus in vitro culture, after 48 h, which also can cause villus infection. Virus can reproduce in CT, EVT, ST, interstitial cells and around villous interstitial substance small blood vessels in villus.

### c-erbB-2 protein expression level

**Figure 3:**
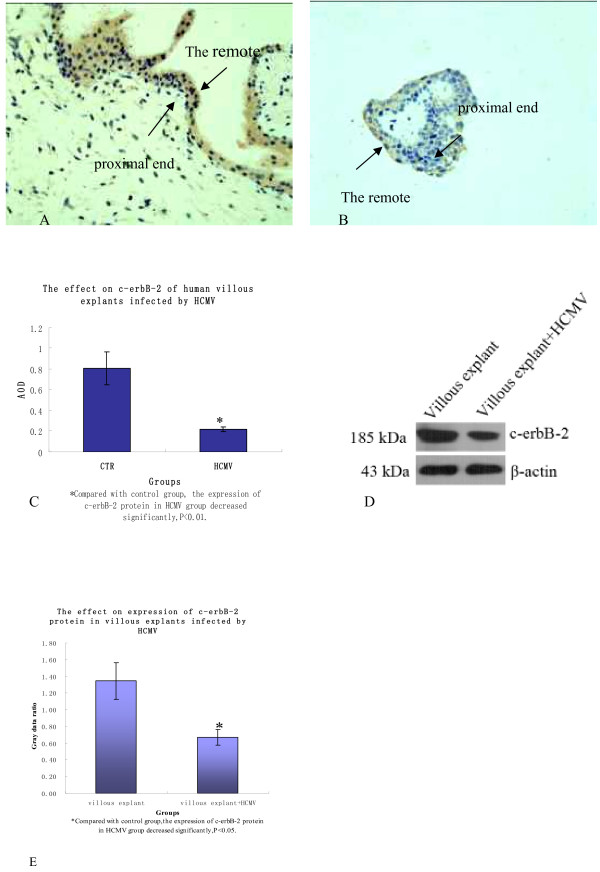
**The expression of c-erbB-2 protein in two groups' villous explants**. A, B. In the villus of two groups, c-erbB-2 protein mainly expressed in ST remote membrane, EVT especially villous stem cell column remote EVT membrane, but little in ST proximal end membrane and interstitial cells; C. According to the comparison of average optical density of two groups' c-erbB-2 protein expression, the protein expression of infected group reduced obviously(P < 0.01); D. Western blot method was used to detect c-erbB-2 protein of two groups' villus and the protein was detected at 185kDa; E. In infected group, c-erbB-2 protein expression level reduced(p < 0.05). Above these experiments were repeated 3 times.

Immunohistochemistry staining results were shown in Figure 3A, B, C, it was found that villous c-erbB-2 protein of control group and infected group expressed in a large quantity in ST remote plasma membrane, EVT, especially villous stem cell column remote EVT membrane, while expressed little in ST proximal end membrane and interstitial cells, and the expression level of infected group reduced(P < 0.01). Western blot method detects the two groups'villus(Figure 3D, E), and c-erbB-2 protein aimed strips can be detected in 185kDa, where infection group's expression level decreased(P < 0.05). The results above indicate that infected HCMV may lead to the decrease of villous c-erbB-2 protein expression.

### MMP-2 and MMP-9 protein expression level

**Figure 4:**
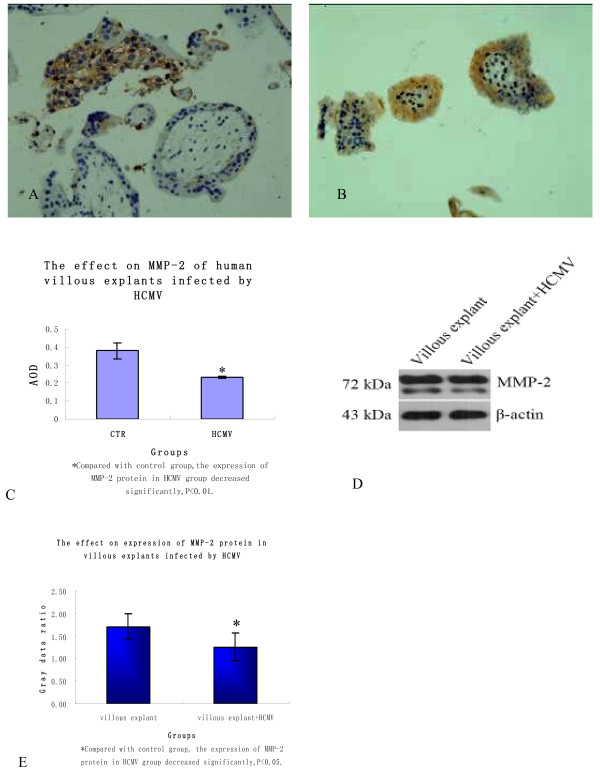
**The expression of MMP-2 protein in two groups' villous explants**. A, B. In the villus of two groups, MMP-2 protein expressed mainly in EVT endochylema and little in VT, ST and interstitial substance; C. According to the comparison of average optical density of two groups' MMP-2 protein expression, the protein expression significantly decreased in infected group(P < 0.01); D. Western blot method was used to detect MMP-2 protein of two groups' villus and the protein was detected at 72kDa; E. In infected group, MMP-2 protein expression level reduced(P < 0.05). Above these experiments were repeated 3 times.

**Figure 5:**
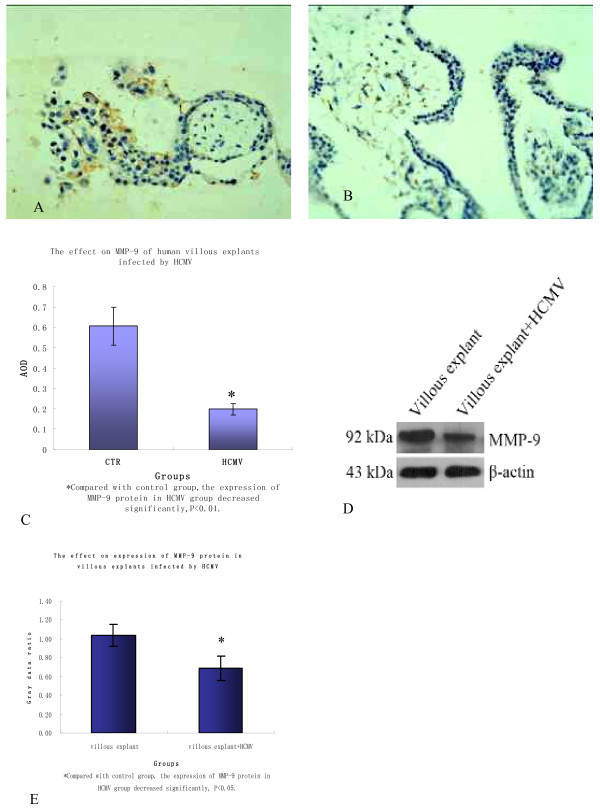
**The expression of MMP-9 protein in two groups' villous explants**. A, B. In the villus of two groups, MMP-9 protein expressed mainly in villous interstitial substance, EVT and VT endochylema but little in ST; C. According to the comparison of average optical density of two groups' MMP-9 protein expression, the protein expression significantly decreased in infected group(P < 0.05); D. Western blot method was detected MMP-9 protein of two groups' villus and the protein was detected at 92kDa; E. In infected group, MMP-9 protein expression level reduced(P < 0.05). These experiments above were repeated 3 times.

Immunohistochemistry staining showed that the two groups' MMP-2 proteins both expressed in EVT endochylema in abundance, but little in VT, ST and interstitial substance(Figure 4A, B); MMP-9 protein expresseed largely in villous interstitial substance, EVT and VT endochylema and little in ST(Figure 5A, B). The expression level of MMP-2 and MMP-9 of infected group both decreased(P < 0.01 and 0.05, the results are shown respectively in Figure 4C and Figure 5C); when Western blot method was used, target strips can be detected at 72kDa and 92kD in the two groups(Figure 4D and Figure 5D), but the two protein expression levels both reduced in infected group(P < 0.05, the results are shown respectively in Figure 4E and Figure 5E). The results above indicate that infected HCMV may lead to the decrease of villous MMP-2 and MMP-9 protein expression.

## Discussion

HCMV is the most common pathogen in intrauterine infection. Among the new born babies in Europe and America, HCMV infection rate is 2%, while that in developing countries is even higher[7]. HCMV intrauterine infection may cause miscarriage, stillbirth, fetus developmental retardation, central nervous system damage and other serious results, but its specific pathogenesis remains unknown. HCMVpp65 protein is the major envelope protein in early stage of HCMV reproduction. The definite symbol of HCMV active infection is controlling HCMV gene expression, inhibiting host's cell metabolism and promoting replication of virus[8].

In the later stage of blastula implantation, CT is divided into VT and EVT. VT locates in inner layer of villus, being active in karyokinesis and able to fuse and form ST which floats on the surface of villus, functioning as internal secretion, immunity and material exchange; EVT proliferates and migrates, gathering together to be cell column and invading into uterus decidua and superficial muscle layer, forming anchoring villus, which enables placenta to be fixed in vascular system of uterus[9]. During embryo implantation, EVT invades decidua and uterus superficial muscle layer and physiologically remodels uterine spiral arteries, which is the necessary condition of successful pregnancy. EVT invasion function decreases, which may lead to miscarriage, stillbirth, fetus developmental retardation, gestational hypertension and other complications of pregnancy. In order to probe the molecular mechanism of HCMV intrauterine infection's causing miscarriage, stillbirth, fetus developmental and other abnormal outcomes, early-pregnancy villus of healthy women in normal pregnancy is used for culturing in vitro, and HCMV is added into culture medium. After 48 h, it is observed that villus HCMV infection condition and the c-erbB-2, MMP-2 and MMP-9's expression levels in villus and characteristics of space distribution change. Immunohistochemistry staining results show that HCMVpp65 antigen signal does not show in control group, but in almost all CT, EVT, ST, interstitial cells and around small blood vessels of villous interstitial substance, that is, after adding HCMV into villous culture medium, 48 h later, villous cells are infected.

c-erbB-2 is part of epidermal growth factor receptor, being a kind of oncogene protein with tyrosine kinase activity and closely linked with embryogenesis, tissue repair and regeneration. It is found that c-erbB-2 highly expresses in EVT which is cultured in vitro, being one of the symbolic proteins of EVT[10-12]. c-erbB-2's expression time and space is highly consistent with EVT invasion function in villus. Therefore, c-erbB-2 can be used to analyze EVT invasion function in villus. This experimental result indicates that c-erbB-2 mainly expresses in early-pregnancy ST remote plasma membrane, EVT, especially villous stem cell column remote EVT's plasma membrane, being consistent with the reports of Mühlhauser et al[13]; HCMV infection brings about the reduce of villous c-erbB-2 expression level, which probably is related with the decrease of EVT invasion function.

MMP is one of the key enzymes which affect EVT migration and invasion function, being characteristic of specificity and timeliness in various trophocytes. Maternal-fetal interface's physical environment, changes in extracellular matrix components, growth factor, cell factor and hormone together maintain the balance of MMP/tissue inhibitor of metalloproteinase(TIMP) system and control the cytotrophoblast cells' proliferation, differentiation and invasion. It is found in study that MMP-2 and MMP-9 are the main stroma metalloprotease which mediate EVT invasion[14]. MMP-9 highly expresses in early and medium pregnancy EVT; MMP-2 expresses in all EVT during the whole gestational period and is active in early and medium pregnancy. The changes of expression level and activity are the same as EVT invasion function's changes[15]. In our study, villous immunohistochemistry staining shows that MMP-2 and MMP-9 protein express largely in early pregnancy villous EVT endochylema, while MMP-9 protein also expresses in high level in early pregnancy villous interstitial substance and VT endochylema; HCMV infection results that villous MMP-2 and MMP-9 expression level both significantly decrease. Villous MMP-2 and MMP-9 expression level reduces, which will lower EVT's proliferation, differentiation and invasion function. The mechanism of HCMV damaging the balance of MMP/TIMP system still remains unclear.

## Conclusions

This paper tentatively proves with in vitro experiments that HCMV can infect almost all CT, EVT, ST, interstitial cells and villous interstitial substance inside villus in vitro; HCMV reproduces and proliferates in early pregnancy villus, arousing villous c-erbB-2, MMP-2 and MMP-9 protein expression level decrease, which may be one of the pathogenesis that HCMV infection causes the reduce of EVT invasion function, miscarriage, stillbirth, cerebellar malformation, fetus developmental retardation and other offspring abnormalities. Next, related researches will be conducted through whole animal experiments.

## List of abbreviations

HCMV: Human cytomegalovirus; CT: cytotrophoblast; VT: villous trophoblast; EVT: extravillous cytotrophoblas; ST: syncytiotrophoblast; MMP: matrix metalloproteinase; TIMP: tissue inhibitor of metalloproteinase; HE: hematoxylin eosin.

## Competing interests

The authors declare that they have no competing interests.

## Authors' contributions

LT performed all experiments and drafted the manuscript. CJJ, YZZ, XJ and ZDD participated in the design of the study and contributed to drafting the manuscript. CSH conceived of the study, and participated in its design and coordination and revised the manuscript. All authors read and approved the final manuscript.

## References

[B1] Hyde TB Schmid DS Cannon MJ Cytomegalovirus seroconversion rates and risk factors: implications for congenital CMV Rev Med Virol 2010 20 311 26 10.1002/rmv.659 20645278

[B2] Takako Yamamoto-Tabata Susan McDonagh Hsin-Ti Chang Human Cytomegalovirus Interleukin-10 Downregulates Metalloproteinase Activity and Impairs Endothelial Cell Migration and Placental Cytotrophoblast Invasiveness In Vitro J Virol 2004 78 2831 2840 10.1128/JVI.78.6.2831-2840.2004 14990702 PMC353759

[B3] Lorenzi T Marzioni D Giannubilo S Expression patterns of two serine protease HtrA1 forms in human placentas complicated by preeclampsia with and without intrauterine growth restriction Placenta 2009 30 35 40 10.1016/j.placenta.2008.10.016 19056122

[B4] Jokhi PP King A Loke YW Reciprocal expression of epidermal growth factor receptor (EGF-R) and c-erbB2 by non-invasive and invasive human trophoblast populations Cytokine 1994 6 433 42 10.1016/1043-4666(94)90068-X 7948752

[B5] Dong M Ding G Zhou J The effect of trophoblasts on T lymphocytes: possible regulatory effector molecules--a proteomic analysis Cell Physiol Biochem 2008 21 463 72 10.1159/000129639 18453754

[B6] Lockwood CJ Oner C Uz YH Matrix metalloproteinase 9 (MMP9) expression in preeclamptic decidua and MMP9 induction by tumor necrosis factor alpha and interleukin 1 beta in human first trimester decidual cells Biol Reprod 2008 78 1064 72 10.1095/biolreprod.107.063743 18276934 PMC3045968

[B7] LaMarca HL Nelson AB Scandurro AB Human cytomegalovirus-induced inhibition of cytotrophoblast invasion in a first trimester extravillous cytotrophoblast cell line Placenta 2006 27 137 47 10.1016/j.placenta.2005.03.003 15921739

[B8] Cristea IM Moorman NJ Terhune SS Human cytomegalovirus pUL83 stimulates activity of the viral immediate-early promoter through its interaction with the cellular IFI16 protein J Virol 2010 84 7803 14 10.1128/JVI.00139-10 20504932 PMC2897612

[B9] Takako Yamamoto-Tabata Susan McDonagh Hsin-Ti Chang Human Cytomegalovirus Interleukin-10 Downregulates Metalloproteinase Activity and Impairs Endothelial Cell Migration and Placental Cytotrophoblast Invasiveness In Vitro J Virol 2004 78 2831 2840 10.1128/JVI.78.6.2831-2840.2004 14990702 PMC353759

[B10] Tarrade A Lai Kuen R Malassiné A Characterization of human villous and extravillous trophoblasts isolated from first trimester placenta Lab Invest 2001 81 1199 211 10.1038/labinvest.3780334 11555668

[B11] Oki N Matsuo H Nakago S Effects of 3,5,3'-triiodothyronine on the invasive potential and the expression of integrins and matrix metalloproteinases in cultured early placental extravillous trophoblasts J Clin Endocrinol Metab 2004 89 5213 21 10.1210/jc.2004-0352 15472228

[B12] Kliman HJ Uteroplacental blood flow: the story of ecidualization, menstuation and trophoblast invasion Am J Pathol 2000 157 1759 1768 10.1016/S0002-9440(10)64813-4 11106547 PMC1885765

[B13] Mühlhauser J Crescimanno C Kaufmann P Differentiation and proliferation patterns in human trophoblast revealed by c-erbB-2 oncogene product and EGF-R J Histochem Cytochem 1993 41 165 73 8093455 10.1177/41.2.8093455

[B14] Ioannidis I Dimo B Karameris A Comparative study of the immunohistochemical expression of metalloproteinases 2, 7 and 9 between clearly invasive carcinomas and "in situ" trophoblast invasion Neoplasma 2010 57 20 8 10.4149/neo_2010_01_020 19895168

[B15] Isaka K Usuda S Ito H Expression and activity of matrix metalloproteinase 2 and 9 in human trophoblasts Placenta 2003 24 53 64 10.1053/plac.2002.0867 12495660

